# An Efficacy Study between High Viscosity Glass Ionomers and Resin Sealants in Fissure Caries Prevention: A 2-Year Split Mouth Randomized Controlled Trial

**DOI:** 10.1055/s-0041-1731925

**Published:** 2021-08-25

**Authors:** Praveen Bhoopathi Haricharan, Sreenivas Voruganti, Arpitha Kotha, Madhuniranjanswamy Mahalakshmamma Shivanna, Bhavana Gandhi, Nanditha Suresh

**Affiliations:** 1 Dental Public Health Unit, Faculty of Dentistry, Asian Institute of Medicine, Science and Technology University, Bedong, Kedah, Malaysia; 2 Department of Public Health Dentistry, Kamineni Institute of Dental Sciences, Telangana, India; 3 Department of Public Health Dentistry, MNR Dental College & Hospital, Telangana, India; 4 Department of Community Dentistry, Penang International Dental College, Butterworth, Penang, Malaysia; 5 Faculty of Dentistry, AIMST University, Bedong, Kedah, Malaysia; 6 Department of Periodontology, Asean memorial dental college and hospital, Tamil Nadu, Chennai, India

**Keywords:** atraumatic restorative treatment, fissure sealants, glass ionomer sealants, resin-based sealants

## Abstract

**Objectives** This clinical trial compared the efficacy of atraumatic restorative treatment (ART) sealants against resin-based sealants in terms of their retention and fissure caries preventive benefits over a period of 24 months among a section of school children in the Southern Indian state of Telangana.

**Materials and Methods** A split mouth clinical trial employed 198 children, who received these sealants on their lower permanent first molars. Retention was assessed 6 monthly and caries annually

**Statistical Analysis** Chi-square tests were utilized to analyze the retention rate and the incidence of dental caries between the two groups. Kaplan–Meier survival analysis plotted the cumulative survival percentage of partially, and fully retained sealants and the survival of dentin carious free pits and fissures among both the groups. A linear binary logistical regression analysis calculated the odds ratio.

**Results** A statistical significant difference was observed in the retention rate between these sealants at every follow-up stage. The cumulative survival percentage of ART and resin sealants was calculated to be 30.9 and 37.5% by the end of 2 years. The Kaplan–Meier analysis showed no significant difference with regard to the survival of dentin carious free pits and fissures. The odds ratio for this trial was 0.747 (95% confidence interval: 0.493–1.13)

**Conclusion** Resin sealants fared better than ART sealants in the field of retention. However, no significant differences were observed with regard to fissure caries prevention by the end of the study period.

## Introduction

Dental caries is the most common disease affecting almost all the communities worldwide with an increased prevalence in school children. It is reported that around 90% of the carious lesions occur on the susceptible occlusal surfaces of the teeth in the school going children age group. 1 2 The fissure sealants have been in use since the 1960s to seal pits and fissures (of the molars/premolars) among individuals with high caries risk. The pit and fissure sealants are considered to be one of the effective methods for preventing dental caries. 3 4

The current management of caries risk individuals involves use of one of the two main groups of sealants, namely the resin and glass ionomer based sealants. 5 The resin sealants are primary treatment modalities considering individuals with high for caries risk, owing to their superior retentive and physical properties. These properties enable them to retain on occlusal surfaces for a considerable period of time or boosting the longevity of restoration. 6 However, the routine use of these resin sealants is limited by technique sensitivity during clinical application procedure. 3

The glass ionomer sealants, on the other hand, are hydrophilic in nature and therefore are less dependent on profound moisture control for retention on tooth surfaces. 7 The introduction of high viscous glass ionomer cements with improved mechanical properties, rapid setting time, and a higher abrasive resistance led them to be used in the atraumatic restorative treatment (ART) framework. The ART involves placing high viscosity sealants onto the pit and fissures, followed by the press finger technique and removal of excess material via hand instruments. These sealants are thus popularly termed as ART sealants. 8 9

The ART sealants employing high viscosity glass ionomers (HVGIC) are reported in literature to have enjoyed a higher survival rate in comparison to conventional low viscosity glass ionomers. 10 11 However, the clinical picture turns ambiguous, the moment ART sealants are stacked up against resin-based sealants, which are considered the gold standard, in the field of fissure caries prevention. A recent systematic review had identified a research lacunae and stated the need for further high quality trails comparing the effectiveness of resin sealants and high viscosity glass ionomer sealants. 12

Considering the lacunae in the literature, a randomized controlled trial was carried out among a section of school going children belonging to a district in the Southern Indian state of Telangana, India. The objective of this trial is to report the efficacy of ART sealants, which employ HVGICs and resin-based sealants in terms of retention and fissure caries prevention over a period of 24 months. The null hypothesis states no difference between these types of sealants in terms of retention and caries preventive benefits.

## Materials and Methods

### Study Design

This study is a randomized controlled clinical trial employing a split mouth design, undertaken to assess the clinical performance of ART sealants in comparison to resin-based sealants. The current trial was registered with the U.S. National Library of Medicine (ClinicalTrials.gov NCT02408601).

### Ethical Aspects

The ethical clearance for the study was obtained from the institutional review board, Kamineni Institute of Dental Sciences (reference number: KIDS/2015/13D), Telangana, India. The trial was carried out in accordance with Declaration of Helsinki and in adherence to the CONsolidated Standards of Reporting Trials (CONSORT) guidelines.

### Study Population

School children belonging to the lower socioeconomic group, in the age group between 6 and 12 years, enrolled in the largest semi-autonomous school in this region were invited to participate in the study. Appropriate permissions had been obtained from the district educational officer and the school headmaster prior to the initial approach.

This district is one of the prominent districts in the newly carved out South Indian state of Telangana and is a known endemic fluoride belt. 13 The municipal drinking water supply of this district is not fluoridated. The usage of fluoridated toothpaste is widespread in this district, and the dental caries prevalence of the 12/15-year-old children in this region was reported to be 56.3%. 14

Only children, whose parents or guardians had given their consent, were clinically examined by the chief investigator to assess the baseline status of mandibular permanent first molars. The examinations were carried out within the school premises by using portable dental chair and standard diagnostic instruments. The presence of caries was decided based on the criteria proposed by the World Health Organization (1987). 15

### Sample Size Estimation

The sample was calculated based on an expected difference of 15% between the two groups, 16 with significance level at 5%, power of the study being 80%, at a two tailed 90% confidence interval and the percentage success in both groups to be 50%, to be 191 per group.

### Inclusion/Exclusion Criteria

The children aged 6 to 12 years, with dentin caries free contra lateral permanent mandibular first molars with a well-defined fissure system, having a Decayed, Missing, and Filled Teeth score >2 and with fully exposed clinical crowns were included for the study. The radiographic examinations were not considered to screen these subjects for presence of carious lesions. The participants with shallow fissure system, those with preexisting cavitated carious lesions on lower permanent molars, with history of allergic reactions toward dental materials, enamel hypoplasia, and those uncooperative were excluded from the study.

### Randomization Procedure

The ART sealants (Ketac Molar Easymix, 3 M ESPE, Seefeld, Germany) and Helioseal (Vivadent, Schaan, Liechtenstein) were compared with each other in this clinical trial. The trial adopted a split mouth design using contra lateral mandibular permanent first molars. The mouth was split vertically into two sides (right and left), and a simple randomization procedure was followed. Within each participant, random numbers were chosen to select which side received either the resin or the glass ionomer material.

### Operator Training

A single graduate student of the department was chosen for these sealant applications. He was put through theoretical sessions and underwent hands on training session in the simulation lab on the practical aspects of sealant handling and application. The graduate student applied these sealants randomly to a set of 10 children, who were not a part of the study sample. Any errors or discrepancies in the clinical procedures were discussed and corrected appropriately.

### Sealant Applications

The study subjects were summoned to the preventive clinics of the dental institute in a systematic manner for sealant applications. The customary oral prophylaxis was carried out for every child prior to the sealant placement with an ultrasonic scaler, followed by polishing with slurry water and rotating brush. The isolation was achieved by using cotton rolls and saliva ejector. The sealant applications were carried in preventive dental clinics of the teaching institute, with active supervision from the Department of Public Health Dentistry.

The interventional procedure for resin sealants group involved isolating the specific molar tooth, acid etching (with 35% phosphoric acid for 15–20 seconds), followed by washing with water. The tooth was then air dried by using a three-way in one syringe. The resin sealant was introduced subsequently and light cured accordingly. With regard to the ART group, the teeth were conditioned with 10% polyacrylic acid for 10 to 15 seconds and cleaned by cotton pellets. The glass ionomer material was hand mixed and placed onto the occlusal surface by using the finger press technique. 17 The retention of the sealants were evaluated, and occlusal discrepancies were corrected by using an articulating paper. The participants and their guardians were instructed to follow postoperative guidelines following the placement of sealant material.

### Postoperative Evaluation

The participants were followed up at the end of the 6 months, 1 year, 1.5 years, and 2 years, respectively. These evaluations were carried out by the chief examiner who had been involved in the baseline examination of the study subjects prior to the start of the study.

The retention of the sealants and the presence of caries were assessed at each of the designated follow-ups within the school premises. A sharp sickle shaped explorers were employed to assess the retention and community periodontal probes were used to diagnose carious lesions that developed over time. The retention of the sealants was graded according to Simonsen’s criteria, 18 which categorized sealant retention into completely retained, partially retained, and completely lost. The occurrence of a caries lesion was considered if the depth of the lesion involved the dentin as stated by the World Health Organization. 15

Duplicate examinations were carried out at the scheduled follow-up intervals on about 10% of the sample selected randomly to reassess retention and dental caries characteristics. The intraexaminer reproducibility of this examiner was monitored appropriately.

### Data Analysis

The data were coded appropriately and entered into Microsoft Excel worksheet and analyzed by using the SPSS software version 20 (IBM, Armonk, New York, United States). The intraexaminer reliability for the initial baseline screening and subsequent follow-ups calculated by means of kappa value. Significance was considered when the *p* -values were less than 0.05 in all instances. The Chi-square tests were employed to assess the retention rate and the caries preventive benefits between the materials at designated follow-up periods. The Kaplan–Meier survival analysis was performed to assess the cumulative retention rate and the survival of dentin caries free pit and fissures amongst both groups, with long-rank tests employed to ascertain the significance levels. The hazards function graph was presented to depict the risk of sealant loss over a period of time. A binary logistic regression analysis was performed to calculate the odds of developing caries amongst the two groups.

## Results

**Fig. 1:**
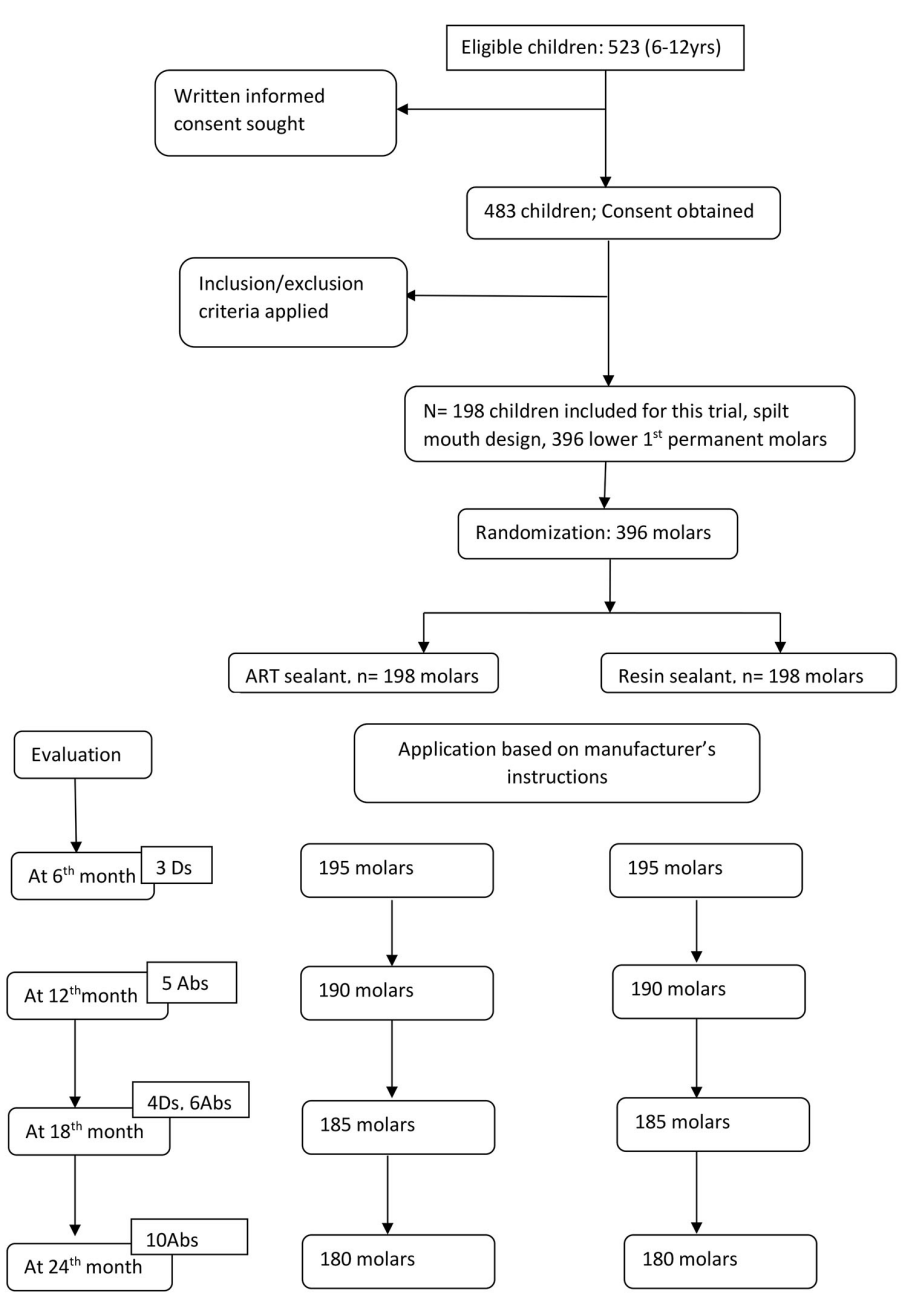
Consort flow chart representing the study design.

The study population included the entire cohort of children in the age group between 6 to 12 years, which stood at 523. Parents/guardians of 40 children refused to let their children to be a part of this trial. After matching the rest against the inclusion and exclusion criteria, 198 children (103 males, 95 females), with an mean age of 9.5 ± 2.21 years were included in the study. Each participant had received both sealants under study, totaling to 396 sealant applications for all the 198 children. There were 7 dropouts and 21 absentees at various stages of the study period ( Fig. 1 ). The intraexaminer agreement values for baseline examination and sealant assessment was 0.93 and 0.92, respectively.

The retention rates of these sealants at designated intervals are represented in Table 1 . A marginal difference in retention rates has been observed at the 6 months. At 12th month, about one-fourth of the ART sealants were completely lost, with 8.4% of the resin sealant being lost totally. More than half of the ART sealants were completely lost at the end of the study period, with about 30% total loss seen in the resin group. The analysis reveals a statistically significant difference between their retention rates at each of the designated intervals. With regard to carious lesion formation on these sealed surfaces, a substantial percentage of occlusal surfaces have remained carious free across both groups of sealants, with the incidence of dental caries being 10.5 and 6.66% for ART and resin group, respectively with no significant difference observed in this domain ( Table 2 ).

**Table 1:** Frequency distribution of retention rates at each of the designated follow-up intervals among atraumatic restorative treatment and resin sealants

		ART sealant group	Resin-based sealant	*p* -Value ^a^
At 6 mo	Completely retained	147 (75.3%)	163 (83.5%)	0.039
	Partially retained	37 (18.9%)	27 (13.8%)	
	Completely lost	11 (5.6%)	5 (2.56%)	
At 12 mo	Completely retained	112 (58.9%)	139 (73.1%)	0.001
	Partially retained	32 (16.8%)	34 (17.8%)	
	Completely lost	46 (24.2%)	17 (8.9%)	
At 18 mo	Completely retained	72 (38.9%)	98 (52.9%)	0.000
	Partially retained	34 (18.3%)	53 (28.6%)	
	Completely lost	79 (42.7%)	34 (18.3%)	
At 24 mo	Completely retained	57 (31.6%)	71 (39.4%)	0.000
	Partially retained	26 (14.4%)	54 (30%)	
	Completely lost	97 (53.8%)	55 (30.5%)	
Abbreviation: ART, atraumatic restorative treatment. ^a^ McNemar’s test.				

**Table 2:** Comparison of caries incidence between teeth sealed with atraumatic restorative treatment and resin sealants

		ART sealant group	Resin sealant group	*p* -Value ^a^
At 12 mo	Caries absent	182 (95.7%)	185 (97.3%)	
	Caries present	8 (4.21%)	5 (2.63%)	0.210
At 24 mo	Caries absent	161 (89.4%)	168 (93.3%)	
	Caries present	19 (10.5%)	12 (6.66%)	0.265
Abbreviation: ART, atraumatic restorative treatment. ^a^ McNemar’s test.				

**Fig. 2:**
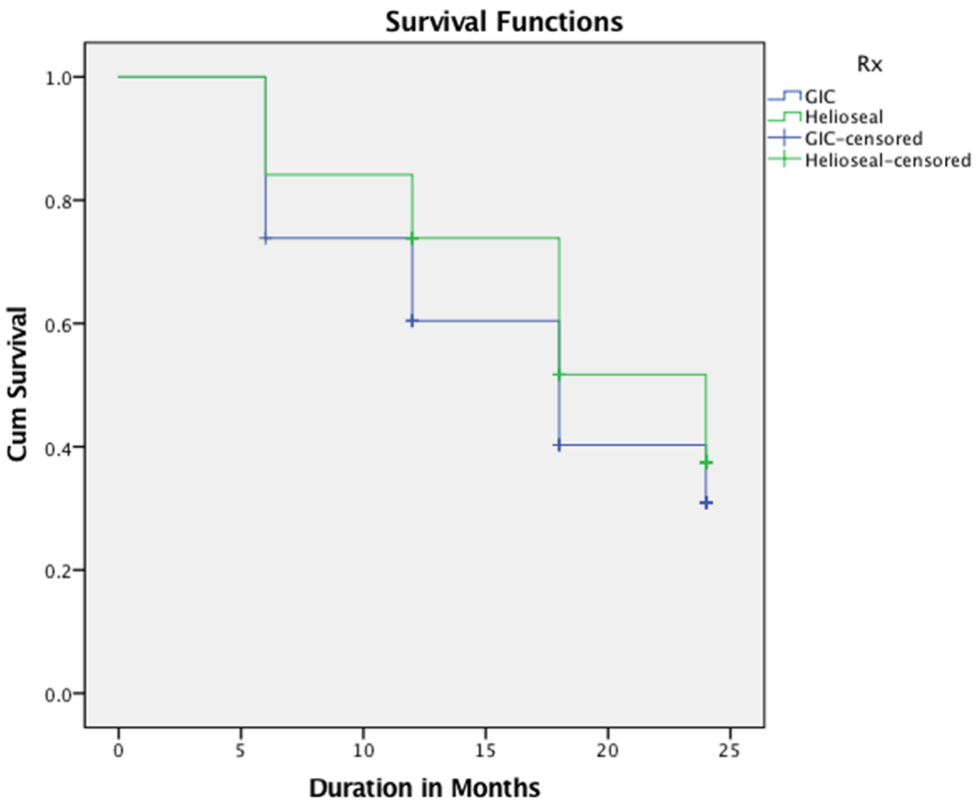
Kaplan–Meier survival analysis of partial and fully retained sealants over a period of 24 months, log-rank test (Mantel Cox test), *p* = 0.030.

**Fig. 3:**
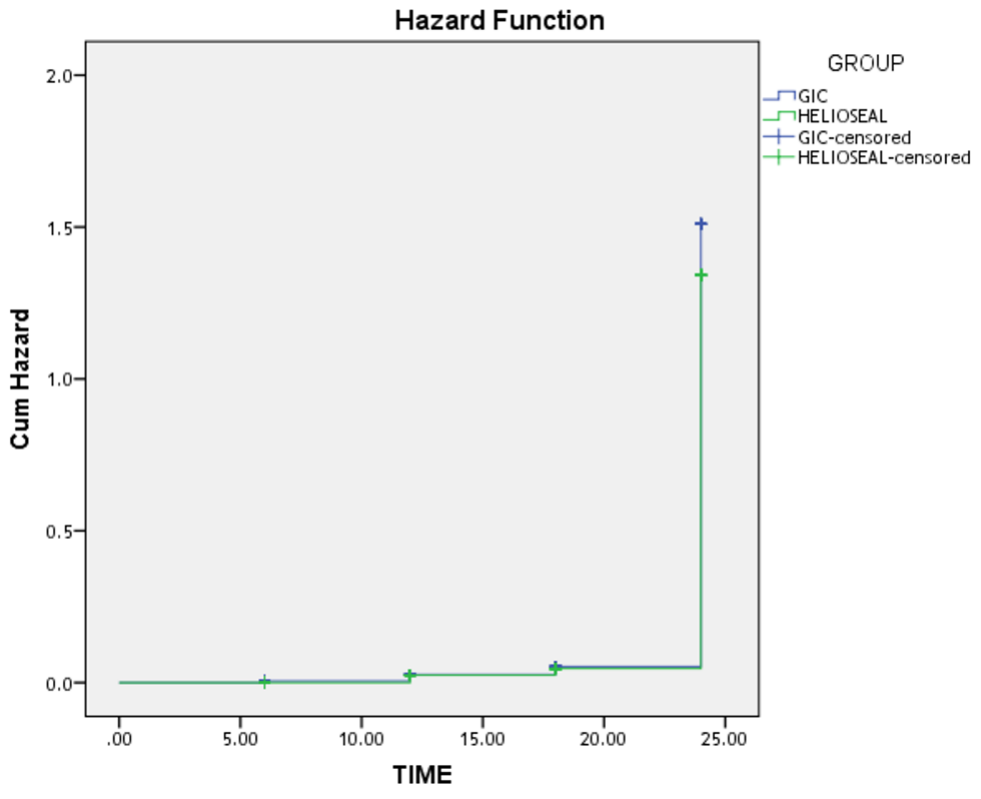
Hazard function graph depicting the risk of sealant loss as observed in the follow-up evaluation.

The Kaplan–Meier survival analysis presented a cumulative survival rate of 37.5% at the end of 2 year period and a median survival time of 24 months with resin sealants. Similarly, ART sealants had a cumulative survival rate of 30.9%, with a median survival time of 18 months ( Tables 3 , 4 ; Fig. 2 ). The Long-rank test showed a statistical difference between cumulative retentive rates of these materials ( Fig. 2 , *p* = 0.030). The Cox regression coefficient for the survival of these sealants was directly proportional, with the *p-* value at 0.070. Analyzing the risk of sealant loss over a period of time, a hazard function graph is depicted, which explicitly states that risk of sealant loss across both groups exponentially increases with time ( Fig. 3 ).

**Table 3:** The mean and median survival time of fully and partially retained sealants in months with regard to atraumatic restorative treatment and resin-based sealants

	Mean ^a^				Median			
			**95% confidence interval**				**95% confidence interval**	
	**Estimate**	**SE**	**Lower bound**	**Upper bound**	**Estimate**	**SE**	**Lower bound**	**Upper bound**
ART	16.473	0.533	15.429	17.517	18.0	1.057	15.929	20.071
Resin	18.578	0.483	17.633	19.524	24.0	1.167	21.712	26.288
Abbreviations: ART, atraumatic restorative treatment; SE, standard error.								

**Table 4:** Cumulative survival percentages and standard error of fully and partially retained resin and atraumatic restorative treatment sealants over a period of 24 months

	Resin sealant			ART sealant			
Interval	**Survival (%)**	**SE**	**95% CI**	**Survival (%)**	**SE**	**95% CI**	***p*** **-Value**
6 mo	84.1	2.6	0.837–0.845	73.8	3.1	0.734–0.742	<0.05
12 mo	73.8	3.1	0.734–0.742	60.4	3.5	0.599–0.609	<0.05
18 mo	51.7	3.6	0.512–0.522	40.3	3.5	0.398–0.408	<0.05
24 mo	37.5	3.5	0.370–0.380	30.9	3.4	0.304–0.314	<0.05
Abbreviations: ART, atraumatic restorative treatment; CI, confidence interval; SE, standard error.							

**Fig. 4:**
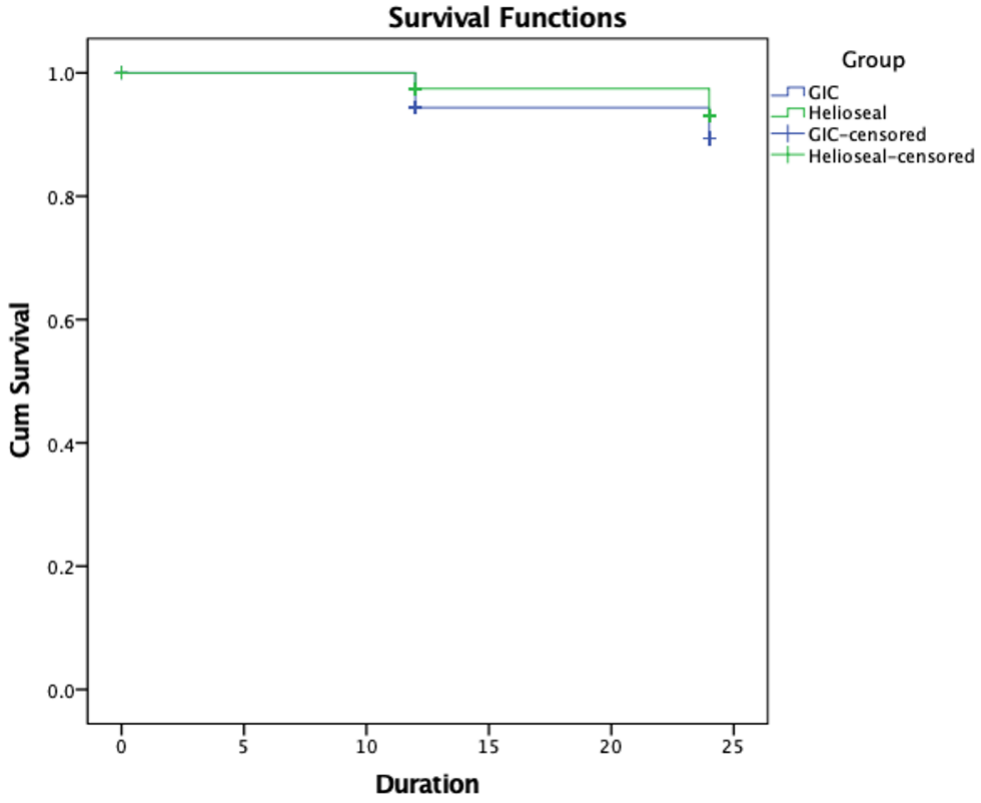
Kaplan–Meier survival analysis pertaining to survival of dentin caries free pit and fissures at 12th and 24th months, respectively, log-rank test (Mantel Cox test), *p* = 0.194.

The Kaplan–Meier survival analysis pertaining to survival of dentin carious lesion free pits and fissures showed no significant difference between these two materials ( Fig. 4 ). A binary logistic regression analysis calculated the odds ratio (OR) to be 0.747 (95% confidence interval: 0.493–1.13).

## Discussion

A randomized controlled split mouth clinical trial was conducted to test the hypothesis pertaining to the performance of ART and resin sealants in participants with pit and fissures of mandibular molar teeth. The initial baseline examination and follow-up evaluations of the school children was considered as accurate, on account of the high intra examiner agreement values that were observed.

The blinding of the operator to these clinical procedures could not be done by owning to the nature of the trial. The lone operator did not have an inclination toward a particular clinical procedure as he had been trained just before the start of the trial. The participants had received these preventive therapies for the first time and thus did not have a prediction toward any sealant material. The risk of detection bias could not be avoided in subsequent follow-up intervals, as both sealants were distinctly different in their clinical appearance. Nonetheless, the extent to which this could have affected the outcome is not known and is difficult to assess.

The data obtained during the study period was coded and presented to the statistician for analysis, with statistical interpretation done following the culmination of the data collection. To add to this, the attrition rate for this clinical trial was minimal (3.5%) and thus assumed to not have affected the final outcome. Taking into account these inherent limitations, the internal validity of this trial can be considered as adequate.

The null hypothesis pertaining to sealant retention stands rejected in the current study. The cumulative survival percentage of ART sealants has been significantly lower in comparison to the resin group at each designated follow-up interval. This is in line with a recently published systematic review. 4 It is imperative to note that ART sealants in our study were placed by a single-trained clinician in a controlled clinical environment rather than an outreach facility, for which it was originally designed for.

Despite the presence of a favorable working environment, the 2-year cumulative survival percentage of ART sealants in this study stood at 30.9%, which was way below in comparison to studies carried out by Vieira et al 19 (99%, 1-year survival), Holmgren et al 20 (98%, 2-year survival), Luengas-Quintero et al 21 (48.8%, 2-year survival), Liu et al 22 (93%, 2-year survival), Hilgert et al 23 (67.7%, 2-year survival), Zhang et al 24 (80.7%, 2-year survival), Zhang et al 25 (98.5%, 2-year survival), and Frencken et al 26 (98%, 2-year survival). A recently published clinical trial also reported a low ART retention rate when applied within school premises. 27 Interestingly, the 2-year cumulative survival percentage of 37.5% with respect to resin sealants in our study is also well below the retention rates as documented in the literature. 4 6

A holistic perspective into the survival percentages of ART sealants indicates a possible association between clinical experience of operators and retention of these sealants. 26 28 It is a well-known fact that these clinical application steps are meticulous in nature and rely to a large extent on the handling of these materials. The lower cumulative survival percentage observed in our study could well be attributed to the inexperience of the graduate student, despite imparting adequate training prior to the commencement of the study.

The only probable justification for using an inexperienced operator is that for such large-scale preventive programs, the availability of experienced dentists is a difficult proposition in countries lacking adequate oral health care services. However, overzealous analysis of “retention factor” of sealants must be interpreted with caution. It is very common in fissure caries clinical trials to use “retention” as a surrogate end point for caries occurrence or absence, as it makes the conduct of these trials simpler, shorter, and inexpensive. This concept has been invalidated in due course of time and thus clinical recommendations or guidelines inclined toward a sealant with superior retentive properties should be reconsidered. 29

Now that the onus is firmly on fissure caries prevention, the null hypothesis pertaining to this parameter is accepted, as there has been no significant difference between dentin caries preventive benefits between these sealants. Also, no difference was noted in cumulative survival rates of caries free pits and fissures when sealed with ART and resin sealants at the 12th and 24th month, respectively. This is in tune with a recent systematic review 12 and other similar studies conducted by Chen et al, 30 Oba et al, 31 Zhang et al, 25 and Liu. 22 A single study carried out in Syria using a parallel group design found that ART sealants prevented dental caries significantly better than resin sealants over a 1- to 3-year period. 32

It is an established scientific fact that presence of a constant level of fluoride within the oral environment decreases the incidence of dental caries. 33 The study participants reside in an endemic fluoride belt and consume drinking water, which is naturally fluoridated. Concurrently, ART and resin sealants employed here are also fluoride depots and release free fluoride ions into the oral environment and thereby further contribute to the anticariogenic effect. To extent to which these factors could have downplayed the difference between the dental caries lesion preventive effects between these materials is not known. Moreover, in teeth sealed with glass ionomers, evidence clearly pointed out to the presence of remnants of glass ionomer particles in the deeper layers of fissures, even in surfaces with partial or total loss of glass ionomer sealants, thereby buttressing the anticariogenic effect. 34 In a nutshell, the consumption of naturally fluoridated water, the widespread use of fluoridated toothpastes, and fluoride release from sealants may have influenced the true caries preventive differences among the sealants, as well as contributing to anticariogenic effects.

Highlighting the drawbacks of this trial, the use of a split mouth study approach makes it imperative to select a child with at least a pair of caries free permanent first molars, in our case, a pair of caries free contra-lateral lower first permanent molars. This is surely bound to generate selection bias, as children in the high caries risk category may have had developed caries in one of the lower permanent molars, therefore rendering them ineligible for the trial. 35 Additionally, the baseline clinical examination of the children for the presence of caries was done in accordance with the WHO criteria, 15 which detects cavitated lesions extending into the dentin. The use of precise caries detection methods such as diagnodent 36 might have resulted in a greater number of children being excluded from the trial, as it is designed to pick up non cavitated dentin carious lesions. Similarly, radiographic investigations to detect carious lesions were not performed at baseline and at subsequent evaluations.

All the above methods were not engaged in this trial due to lack of adequate resources. The future directions from this study are a well-designed parallel study design and well matched in its baseline characteristics, with children belonging to the high caries risk category in the age group between 6 and 7 years of age, without the presence of “fluoride” confounding factor may reveal the true preventive benefits, in this regard. The comparison of the economic factors of these sealants needs to be assessed, for effective use in community prevention programs.

## Conclusion

The study showed, within its limitations, that no significant differences existed with respect to caries preventive benefits between the resin and glass ionomer pit and fissure sealants. However, in the field of retention, resin sealants fared better than ART sealants in this study population.
